# Editorial: New Advances in Neurorehabilitation

**DOI:** 10.3389/fneur.2019.01090

**Published:** 2019-10-17

**Authors:** Stefano Tamburin, Nicola Smania, Leopold Saltuari, Volker Hoemberg, Giorgio Sandrini

**Affiliations:** ^1^Department of Neurosciences, Biomedicine and Movement Sciences, University of Verona, Verona, Italy; ^2^Neuromotor and Cognitive Rehabilitation Research Centre, University of Verona, Verona, Italy; ^3^Research Unit for Neurorehabilitation South Tyrol, Bolzano, Italy; ^4^Department of Neurology, State Hospital Hochzirl, Zirl, Austria; ^5^Department of Neurology, Stiftung Rehabilitation Heidelberg Gesundheitszentrum Bad Wimpfen GmbH, Bad Wimpfen, Germany; ^6^Neurorehabilitation Unit, Istituto di Ricovero e Cura a Carattere Scientifico C. Mondino Foundation, Pavia, Italy; ^7^Department of Brain and Behavioral Sciences, University of Pavia, Pavia, Italy

**Keywords:** headache, multiple sclerosis, neurorehabilitation, pain, Parkinson's disease, rehabilitation, robot therapy, stroke

The face of neurorehabilitation has progressively changed in recent years. Traditional neurorehabilitation procedures may have limited efficacy in most patients with common neurological diseases, such as stroke, Parkinson's disease, spinal cord injury, severe brain injury, spasticity, and cognitive disorders. New technologies have been reported to enhance the effectiveness of rehabilitation strategies in these conditions. They include robotic-assisted training, virtual reality, functional electrostimulation, non-invasive brain stimulation (NIBS) to enhance the intensity and quality of neurorehabilitation, and to manipulate brain excitability and plasticity, as well as innovative approaches such as assistive technology and domotics.

The exploration of the effects of neurorehabilitation technologies and NIBS on plasticity through the use of advanced technologies (i.e., functional MRI, near infrared spectroscopy, high-density EEG, etc.) may represent a surrogate outcome measure in the near future. On the other hand, translational and back-translational models are important to offer robust neurobiological grounds to current rehabilitative approaches to neurological disorders.

The correlation between central nervous system lesions to clinical features and outcomes represents the basis for personalized medicine in neurorehabilitation, a promising perspective to explain the different individual response to the treatment, and to improve the quality of care. The definition of new approaches to the acute and chronic phase of neurological diseases and the most appropriate timing play a key role to optimize neurorehabilitation interventions. Moreover, new randomized controlled trial designs aimed to explore the role of combined drug and physiotherapy treatment are emerging.

Finally, despite for many years evidence-based medicine was, to some extent, far from the field of neurorehabilitation, the interest for systematic reviews, meta-analyses, and consensus conferences is increasing.

The Research Topic “New Advances in Neurorehabilitation” included 20 high-quality manuscripts that offer an interesting scenario on these technological and methodological advances, as well as new features and approaches to neurorehabilitation.

Motor outcome after stroke is traditionally one of the main topics in neurorehabilitation (1), because of the high prevalence of chronic stroke.

Schulz et al. explored whether prefrontal-premotor connections are related to residual motor function in 30 well-recovered chronic stroke patients and 26 controls. The Authors reconstructed direct fiber tracts from dorsolateral and ventrolateral prefrontal cortex to dorsal and ventral premotor cortex, supplementary motor area and the primary motor cortex. Prefrontal-premotor tracts were traceable in both groups. In gross anatomic topography, stroke patients presented only marginal microstructural alterations of these tracts, predominantly of the affected hemisphere. However, there was no significant association between tract-related microstructure of prefrontal-premotor connections and residual motor function after stroke.

Chen et al. studied functional cortico-muscular coupling to evaluate motor function in a pilot study on 8 stroke patients and 8 controls. They quantified the functional connection between electroencephalogram and electromyogram from a hand muscle during steady-state grip task and documented that the multiscale and directional characteristics of cortico-muscular coupling may be disrupted in stroke.

van Duijnhoven et al. tested whether a 5-week perturbation-based balance training program on a movable platform may improve reactive step quality in chronic stroke in a proof-of-concept open study on 20 patients. Despite the absence of a control group, patients ameliorated after the treatment, and improvement was retained after 6 weeks.

Ye et al. assessed the effects of oropharyngeal muscle exercises in a randomized controlled trial (RCT) including 50 stroke patients with moderate obstructive sleep apnea syndrome, of whom 25 were allocated to the active group and 25 to the control group who underwent sham therapy of deep breathing. The obstruction severity measures by polysomnography, patient reported outcome, and anatomic structural remodeling of the pharyngeal airways improved after 6 weeks of active treatment.

Recovery after spinal cord injury (SCI) is one of the hot topics in neurorehabilitation research, because of the young age and the severe impairment in many patients (2).

Zeng et al. explored the role of sorting nexin 27 (SNX27), an endosome-associated cargo adaptor that was found to be involved in many neurological diseases, in a mouse model of SCI. The results of the study suggested down-regulation of SNX27 to be a potential therapy targeting acute neuronal death and chronic neuroinflammation, and promoting nerve repair after SCI.

Schneider et al. studied the reliability of wearable sensor-derived measures of physical activity in 63 wheelchair-dependent SCI patients of different age ranges and level/severity of injury. Activity counts showed consistent high single-day reliability, while measures considerably varied depending, with decreased movement quantity and increased movement quality with rehabilitation progress. The results of the study may be helpful for sensor-based assessments of physical activity in clinical SCI studies.

van Dijsseldonk et al. tested a treadmill task in a virtual environment to improve gait and dynamic balance capacity in 15 incomplete SCI patients in an uncontrolled study. Walking speed, stride length, anterior-posterior gait stability and balance confidence improved, while stability measures in medial-lateral direction was unchanged in the 10 patients who completed the study.

Neurorehabilitation plays a key role in multiple sclerosis (MS) patients, who may complain of motor, sensory, cognitive impairment, and pharmaco-resistant pain (3).

van Beek et al. presented the study protocol for a RCT to investigate the effectiveness of a challenging tablet app-based home-based training intervention to improve dexterity in MS patients. They hypothesized that the program will improve dexterity in the short- and long-term, and that the improved finger and hand functions may generalize to improved activities of daily living and quality of life.

Patients with Parkinson's disease (PD) complain of a wide range of motor and non-motor symptoms, and neurorehabilitation procedures are frequently used together with pharmacological treatment in these patients (4).

Berra et al. reviewed data on body weight support combined with treadmill on PD gait and reported data from an RCT on 36 PD patients. Both active and control groups experienced significant improvement in motor and gait outcomes, but the intragroup analysis documented improvement of cadence and stride duration in the active group and of the swing/stance ratio in the control group. Four patients with chronic pain or anxious symptoms did not tolerate body weight support, which may be an option in case of severe postural instability, balance disorder, and orthostatic hypotension.

Rehabilitation of neuropsychological deficits is an emerging field of research and some papers of the Research Topic dealt with cognitive disorders (5).

Zucchella et al. reviewed, with a narrative approach, current evidence on non-pharmacological treatment (NPT) in the treatment of Alzheimer's disease (AD) and dementia. They concluded that, although NPT is often applied in the multidisciplinary approach to AD and dementia, supporting evidence is still preliminary, and suggested well-designed RCTs with innovative designs, and further studies to offer robust neurobiological grounds for NPT, and to examine the cost-efficacy profile.

Pazzaglia and Galli presented a rehabilitative perspective focusing on the possibility of action observation as a therapeutic treatment for patients with apraxia. They also outlined impacts on neurorehabilitation and brain repair following the reinforcement of the perceptual-motor coupling. This perspective might play a role for future interventions based primarily on action observation in patients with apraxia.

Jang and Seo reported a mini-review on diffusion tensor tractography studies on mechanisms of recovery after injury of the anterior cingulum, a major structure in the limbic system, which is involved in various cognitive functions, including memory, attention, learning, motivation, emotion, and pain perception. Despite most of the reviewed studies were case reports, they indicated that diffusion tensor tractography might be useful for the neurorehabilitation of patients with anterior cingulum injury.

Fabbri et al. presented a study protocol for an RCT exploring the effect of a multi-dimensional tele-rehabilitation program through a user-friendly web application in patients with mild cognitive impairment and vascular cognitive impairment. The proposed tele-rehabilitation program includes cognitive, physical, and caregiver-supported social activities to promote and preserve an active lifestyle and counteract cognitive decline.

Pain has been recognized as a common problem in patients undergoing neurorehabilitation (6), but its impact on rehabilitative procedures and the best treatment practices have been largely not explored (7).

Castelnuovo et al. reviewed the role of the placebo effect for pain relief in neurorehabilitation as part of the recommendations of the Italian Consensus Conference on Pain in Neurorehabilitation (8, 9). The Authors found that placebo treatments showed weak effects in central neuropathic pain, moderate effects in postherpetic neuralgia, diabetic peripheral neuropathy, pain associated to HIV, pain due to fibromyalgia and migraine and weak short-term effects in complex regional pain syndrome. They recommended knowledge of placebo mechanisms to shape the doctor-patient relationship, to reduce the use of analgesic drugs and to train the patient to become an active agent of the therapy.

Falsiroli Maistrello et al. reported a systematic review and meta-analysis of RCTs to establish the effectiveness of manual trigger points treatment compared to minimal active or no active interventions in adults with primary headaches. Based on 7 RCTs, they concluded that manual trigger points treatment of head and neck muscles may reduce frequency, intensity, and duration of attacks in tension-type headache and migraine, but the quality of evidence was very low for the presence of few studies, high risk of bias, and imprecision of the results.

Patients with lesions of the peripheral nervous system frequently undergo neurorehabilitation and, among them, those with brachial plexus lesions are those with the most severe impairment.

Ramalho et al. explored bilateral sensory function in 17 patients with unilateral brachial plexus lesions. The Authors found reduced touch threshold not only in the limb with brachial plexus injury, but also in the contralateral upper limb, where no nerve damage was documented. They interpreted these findings as related to a superordinate model of representational plasticity occurring bilaterally in the brain after a unilateral peripheral injury.

The recent literature suggested that, by combining traditional rehabilitation techniques with new technological approaches, e.g., neuromodulation, biofeedback recordings, novel robotic and wearable assistive devices, the amount of recovery might improve in comparison to traditional treatments (10). Some contributions of the Research Topic dealt with robotic rehabilitation in upper-limb stroke and MS patients.

Takebayashi et al. presented the protocol for a multi-center parallel group prospective, randomized, open-label, blinded endpoint trial on 120 chronic stroke patients with upper-limb motor impairment. Patients will be randomly allocated to three different rehabilitation protocols, namely robotic therapy, standard rehabilitation combined with self-training, or robotic therapy combined with constraint-induced movement therapy.

Wu et al. reported an admittance-based patient-active control scheme for real-time intention-driven control of a powered upper limb exoskeleton, detailing the major mechanical structure, the real-time control system of the robot, the dynamic characteristics of the human-exoskeleton system, and an integrated audiovisual game-like interface. They also reported data from an experimental investigation on 3 healthy subjects and 8 stroke patients to validate the feasibility of the proposed scheme for patient-active rehabilitation training.

Buchwald et al. explored the extent to which robotic arm rehabilitation for chronic stroke may promote recovery of speech and language function in 17 individuals with hemiparesis and aphasia, by pairing intensive robot assisted therapy with sham or active transcranial direct current stimulation (tDCS). The authors found that subjects significantly improved on measures of motor speech production after robot therapy, but active tDCS was not associated with greater gains than sham tDCS, suggesting further investigation into the role of tDCS in the relationship of limb and speech/language rehabilitation.

Gandolfi et al. compared the effect of high-intensity robot-assisted hand training to standard rehabilitation on upper limb recovery and muscle activity in 44 MS patients in a single-blinded RCT. The Authors found no significant between-group differences in primary and secondary outcomes, however, robot-assisted training demonstrated remarkable effects on upper limb use and muscle activity.

Because of the growing interest in neurorehabilitation, documented by the large number of manuscripts submitted to the Research Topic, Frontiers in Neurology opened a new Neurorehabilitation section, to which all researchers interested to the topic may contribute.

## Author Contributions

All authors conceived and designed the study. ST drafted the manuscript. NS, LS, VH, and GS critically revised the manuscript for important intellectual content. All authors approved the final version of the manuscript.

### Conflict of Interest

The authors declare that the research was conducted in the absence of any commercial or financial relationships that could be construed as a potential conflict of interest.
